# Gut Microbiome as a Lifestyle Risk Factor Associated with Prostate Cancer

**DOI:** 10.1016/j.euf.2026.01.001

**Published:** 2026-02-18

**Authors:** Michael A. Liss, James R. White, Molly Doris, Zhao Lai, Teresa L. Johnson-Pais, Robin J. Leach, Martin Goros, Jonathan Gelfond, Brian Wickes

**Affiliations:** a Department of Urology, University of California San Diego, CA, USA; b Department of Surgery, South Texas Veterans Healthcare System, San Antonio, TX, USA; c Resphera Biosciences, Baltimore, MD, USA; d Department of Urology, University of Texas Health Sciences Center, San Antonio, TX, USA; e Department of Molecular Medicine, Greehey Children’s Cancer Research Institute, University of Texas Health, San Antonio, TX, USA; f Departments of Cell Systems & Anatomy and Pediatrics, University of Texas Health, San Antonio, TX, USA; g Department of Population Health Sciences, University of Texas Health, San Antonio, TX, USA; h Department of Microbiology, Immunology & Molecular Genetics, University of Texas Health, San Antonio, TX, USA

**Keywords:** Biomarker, Cancer risk prediction, Fecal microbiota transplantation, Gut microbiome, Inflammatory bowel disease, Machine learning, Microbiome analysis, Personalized medicine, Prostate Cancer Risk Insight using Microbiome, UnderStanding signature, Prostate cancer

## Abstract

**Background and objective:**

Most prostate cancer prevention strategies suggest lifestyle modifications, which lack personalization. Gut microbiome is increasingly recognized as an influencing factor in nongastrointestinal cancers, including prostate cancer. The use of gut microbiome as a lifestyle biomarker could help identify individuals with lifestyle more prone to prostate cancer and allow for modification. We aimed to develop a gut microbiome–based biomarker derived from patients undergoing prostate cancer screening.

**Methods:**

We assessed whether the future cancer risk can be evaluated based on a microbiome risk analysis. After extracting DNA, sequencing, and performing a bioinformatics analysis, we identified 39 unique microbial genera of importance. We utilized an artificial intelligence model to calculate their presence, abundance, and weighted significance, generating a microbiome score (Prostate Cancer Risk Insight using Microbiome UnderStanding [PRIMUS]) that ranges from 0 to 1.

**Key findings and limitations:**

Men with an increasing PRIMUS signature showed a sequential increase in prostate cancer risk. The prostate cancer risk persisted after a median follow-up of 4.5 yr. As a risk-assessment tool, the microbiome score compared favorably with prostate cancer risk calculators. Study limitations include the use of two patient groups to diversify the population for both a screening and a prebiopsy scenario; however, the cohorts used different collection methods, including stool, rectal swabs, and glove tip samples, but the same DNA isolation and sequencing. We relied on the longitudinal approach to help reduce these initial differences.

**Conclusions and clinical implications:**

The gut microbiome may serve as a lifestyle risk factor for prostate cancer, but it is not intended to guide biopsy decisions. The implications of this study hinge on the potential for modifiability of the microbiome that could be tested in future clinical trials on prostate cancer risk reduction.

## 1. Introduction

An imbalance in microbial species reduction in microbial diversity within the gut and specific species of microbes have been linked to cancer formation outside the gut [1]. Microbial changes vary in intensity, is often related to dietary habits and obesity, and has been associated with prostate cancer (PCa) [2].

An illustrative example connecting gut microbiome with PCa was reported in patients with inflammatory bowel disease (IBD), who show a four-fold increased risk of developing clinically significant PCa (csPCa) [3,4]. Multiple studies have demonstrated the importance of lifestyle as a risk factor for PCa; however, there have been limited objective measures of lifestyle assessments linked to PCa. The impetus for this study originates from our preliminary research on the microbial metabolic pathways associated with PCa [5].

We aimed to identify microbial patterns and develop a machine learning–based predictive model for clinical applications in assessing lifestyle factors associated with cancer risk. The primary study objective was to create a microbiome signature associated with PCa that can be utilized as a biomarker to guide lifestyle risk behaviors and not as a biomarker to direct biopsy decisions.

## 2. Patients and methods

### 2.1. Study design

We conducted a prospective observational study between February 1, 2016 and April 19, 2023, at the University of Texas Health San Antonio and the South Texas Veterans Healthcare System at Audie L. Murphy Veterans Hospital. We enrolled the participants and obtained written consent from them through the San Antonio Biomarkers of Risk (SABOR) cohort (IRB#: HSC20000030H). We requested samples as part of a genitourinary repository (IRB#: HSC20050234H) that were collected before prostate biopsy procedures. SABOR represented a screening population with annual follow-up. The prebiopsy cohort underwent rectal swab for fecal collection prior to a for-cause biopsy as part of their clinical care, and an electronic medical record was maintained through the repository using an honest broker system. Data collection and follow-up occurred in parallel during the study period. Full cohort descriptions are provided in the Supplementary material.

### 2.2. Study outcomes

The primary outcome was csPCa, defined as grade group ≥2 (Gleason 3 + 4). The primary predictor was the microbiome score. The secondary outcome was diagnosis of any-grade PCa to include low-grade disease (grade group [GG] 1).

### 2.3. Sample size and power calculation

We estimated the power for each taxon for a range of proportional shifts in the average relative abundance. We expected to find increased statistical power with higher abundance genera; therefore, we focused on the power results for lower-frequency organisms, which reflected an average of 0.25–0.5% and 0.5–1.0% of taxonomic 16S sequences in the gut microbiome. Among the taxa found at this low level, if we assume equal variance in two normally distributed treatment populations and require a significance level of α = 0.05, we expect that with at least 200 samples per group (noncancer vs cancer), we would be able to detect a relative increase of a factor of 3 (such as 0.25% mean abundance increased to 0.75% or 0.5% increased to 1.5%) with statistical powers of 84.8%, 93.8%, and 99.7% for taxa with abundances of 0.25–0.5%, 0.5–1.0%, and 1.0–5.0%, respectively.

### 2.4. Statistical analysis

We utilized the nonparametric Mann-Whitney U test for continuous variables and chi-square test for categorical variables to compare the demographics and the Prostate Cancer Risk Insight using Microbiome UnderStanding (PRIMUS) signature of the two cohorts (biopsy and SABOR cohorts). We then compared the outcomes of the four groups (GG2+, GG1, negative biopsy, and low-risk groups) using the nonparametric Kruskal-Wallis test. Multivariate logistic regression analyses were performed on the standard predictor variables of cancer, including the PRIMUS signature. We used a ten-fold cross-validation to calculate performance statistics. Cross-validation usually outperforms the training and testing sets. Therefore, we randomly divided our cohort into the training (two-thirds) and testing (one-third) sets to evaluate the model’s performance on a complete held-out set. Sensitivity, specificity, and the area under the curve (AUC) were used to compare model performance. We calculated the ideal cutoff value using the Youden index [6]. Time-to-event data were analyzed using the nonparametric log-rank test to determine differences between the high and low scores of the PRIMUS signature, which are displayed in a Kaplan-Meier graph showing a cumulative risk.

### 2.5. PRIMUS signature

After microbial DNA isolation, the data were prepared for taxonomic assignment to analyze their relative association with cancer (Supplementary material). Taxa identified as significant across group comparisons (*n* = 39) were selected for machine learning model training using the random forest method with the *caret* R package (v6.0; R Foundation for Statistical Computing, Vienna, Austria), which employs a ten-fold cross-validation [7]. The receiver operating characteristic (ROC) metric was used for optimization, and the training was tuned with the number of trees (ntree = 500) and *mtry* (the number of predictor variables to randomly select for tree branch growth) set to the floor of the square root of the total input features.

### 2.6. Comparison of PRIMUS and standard of care

We compared the PRIMUS signature with the established PCa risk assessments (such as prostate-specific antigen [PSA] levels and Prostate Cancer Prevention Trial [PCPT] risk calculator scores) to evaluate clinical utility. AUCs were calculated and a decision curve analysis (DCA) was performed for each with bootstrap confidence intervals (CIs). PSA values were obtained via the medical records before specimen collection, typically within 1 mo, considering that all men in the study were undergoing either a prostate biopsy or a PCa screening visit. The PCPT risk calculator version 2 combines age, PSA, family history, race/ethnicity, prior negative prostate biopsy, and digital rectal examination results (https://riskcalc.org/PCPTRC/) [8]. For longitudinal risk assessment, we used the concordance index (c-index) to evaluate the performance of the PRIMUS signature, accounting for clinical variables in a survival prediction model to measure the probability that an individual who was observed to have an earlier event had a higher predicted risk (expected in observed cases). We used a previously published 5-yr PCa prediction model to account for clinical variables [9].

## 3. Results

### 3.1. Study population

We sequenced the DNA isolated from 2022 fecal samples. Our initial population comprised 1270 individuals in the SABOR screening cohort and 752 individuals in the biopsy cohort. After excluding those with poor DNA yields, repeat samples, or repeat individuals, our study cohort included 824 patients (Fig. 1). The groups were combined for an analysis to obtain a large sample size, although the biopsy cohort had an overall higher risk of PCa than the SABOR cohort. The demographic data of these patients were compared based on their cancer status (GG2+, GG1, negative biopsy, and low risk; Table 1). The patients showed significant differences in most categories, including age, prostate examination, race/ethnicity, and family history. The microbial factors included the observed number of microbial species (alpha diversity, *p* = 0.058) and Shannon diversity (beta diversity, *p* = 0.49; Supplementary Fig. 1 and 2). We also compared patient cohorts according to prostate biopsy and race/ethnicity (Supplementary Table 1). We further explored the alpha and beta diversities using principal coordinate analysis plots and used these analyses to develop the PRIMUS signature algorithm (Supplementary Fig. 3).

### 3.2. PRIMUS signature in univariate analysis

We used the entire cohort for a multivariate analysis. No differences were detected in the number of microbial species among the risk groups. The median PRIMUS signature was lowest in the low-risk group and highest in the GG2+ groups (0.216 vs 0.376, *p* < 0.001). According to the univariate analysis, the difference in the PRIMUS signature (any PCa vs no PCa) was significant (*p* < 0.001; Table 1). We divided the PRIMUS signature into quintiles and compared each quintile with the proportion of patients with any cancer or GG2+ PCa, noting a linear and sequential increase in cancer risk in each subsequent quintile (Fig. 2).

### 3.3. Multivariate analysis

We selected a threshold of 0.25 using the Youden index calculation to provide the optimum cutoff point for a binary variable (Supplementary Fig. 4). We then performed a multivariate analysis that controlled for age, race/ethnicity, family history, PSA levels, and abnormal digital rectal examination results, and the PRIMUS signature was identified as a significant predictor in the multivariate logistic regression analyses (odds ratio [OR] 3.5, 95% CI 1.87–6.90, *p* < 0.001; Supplementary Table 2).

### 3.4. PRIMUS signature, PSA, and PCa risk

The PRIMUS test generated an ROC curve AUC of 0.712 (95% CI 0.643–0.769), leading to sensitivity, specificity, positive predictive value, and negative predictive value of 84.8%, 46.9%, 22.2%, and 94.5%, respectively (Fig. 3 and Supplementary Table 3). A similar analysis was performed using anygrade PCa as the outcome (Supplementary Fig. 5 and Supplementary Table 3). The PRIMUS signature correlated with the serum PSA level, the overall PCa prevention trial risk calculator score for cancer risk, and the PCPT score for csPCa (GG2+, *r* = 0.32, 0.29, and 0.32, respectively; all *p* < 0.001). A scatter plot was constructed with the log(s) of the serum PSA concentration on the *y*-axis and the microbiome score on the *x*-axis. The results were weakly correlated, although significant (*r* = 0.12, *p* < 0.001; Supplementary Fig. 6). Next, we performed a DCA using the PRIMUS signature to predict prostate biopsy decision-making and evaluate prediction models [10]. We used risk thresholds ranging from 10% to 35%, which are clinically reasonable for biopsy (treatment) decisions [7]. In this range, the PRIMUS signature provided value for any PCa diagnosis. However, PSA had the most significant net benefit (>35%) near the intersection of the PSA level and PRIMUS signature curves. When comparing DCA for the diagnosis of GG2+ PCa, PSA outperformed PRIMUS for biopsy decision-making (Supplementary Fig. 6).

### 3.5. Longitudinal time-to-event analysis

Fifty-three patients without cancer were recruited into the SABOR cohort and were tested at two time points, approximately a year apart. The microbiome composition did not differ significantly over the evaluated time frame (paired *t* test, *p* = 0.833; Fig. 4); 64% (*n* = 34/53) of the microbiome score values remained below the threshold without intervention over a 1-yr period. We constructed Kaplan-Meier curves for PCa incidence using a PRIMUS signature threshold of 0.25 for the endpoint of GG2+, which was significantly increased for men with a high baseline PRIMUS signature (hazard ratio [HR] 11.1, 95% CI 1.42–87.17, *p* < 0.001; Fig. 4), with a follow-up duration of 4.23 yr (interquartile range [IQR]: 1.70–4.91 yr). We noted similar significant results using any grade of cancer (Supplementary Fig. 7 and 8). The PRIMUS signature remained a significant variable in the Cox regression model accounting for a previously published 5-yr prediction model that included clinical variables of age, PSA, digital rectal prostate examination, family history, and African American race (c-index for the combined model = 0.77, PRIMUS HR 13.1, 95% CI 2.01–89.2, *p* = 0.007; accounting for clinical variables HR 1.03, 95% CI 1.02–1.04, *p* < 0.001).

## 4. Discussion

In this study, we demonstrated that the PRIMUS signature is associated with current and future risks of PCa including csPCa. We demonstrated a biological gradient with increasing PCa risk per decile of PRIMUS. Our findings suggest that the baseline PRIMUS signature threshold of 0.25 is associated with a future PCa risk in men after a median follow-up of 4.5 yr. We used a standard biomarker assessment with AUC and operational characteristics, and followed the participants for a median of 4 yr, attempting to provide a form of validation over time in that men with a high PRIMUS score tended to have a PCa diagnosis over the follow-up period. PSA remained more valuable when deciding whether to perform a biopsy and PRIMUS added minimal benefit when accounting for other clinical factors. Therefore, we anticipate that the PRIMUS test can be used for lifestyle risk profiling with an association with PCa rather than as a tool for making biopsy decisions.

Our study is associational and does not identify a mechanism by which bacteria may increase the PCa risk. However, *Oscillospira* was the most influential bacterium, leading to a lower PRIMUS signature and was known to produce the beneficial short-chain fatty acid butyrate [11,12]. On the contrary, *Peptoniphilus* has a negative influence (increased risk of PCa) and was found in PCa tissue and urine as one of the anaerobic bacteria biomarker sets proposed by Hurst et al [13]. Our findings now implicate that *Peptoniphilus* in the gut microbiome could be a potential source from the gut to colonize the urinary tract. *Enterobacter species* and *Gammaproteobacteria* (*Proteus mirabilis*) produce phenylacetic acid, a microbial metabolite indicator associated with an increased risk of lethal PCa, which also negatively influence the PRIMUS signature [14,15].

We attempted to investigate other additional biases including the sequencing technique (PRIMUS scores generated using two different variable regions, V1–2 vs V3–4), noting a high correlation (Supplementary Fig. 9). We documented no effect from a previous prostate biopsy (Supplementary Fig. 10). The PRIMUS signature does not predict the risk of other cancers (Supplementary Fig. 11). Given the previous consideration that colonized colibactin-producing bacteria may increase the risk of PCa, we investigated the presence of polyketide synthase in conjunction with the PRIMUS signature [16]. While a high PRIMUS score along with colibactin-producing organisms was associated with the highest risk of future PCa, the difference was not statistically significant (Supplementary Fig. 8). We could not link the PRIMUS signature bacteria with other Mendelian randomization studies that identified potential causal bacteria, likely due to heterogeneity and cohorts represented from different countries known to have marked differences in gut microbiome (Supplementary Fig. 12). We collected the baseline and follow-up samples approximately 1 yr later from a subset of 53 men (Fig. 4), showing that 65% remained within their original threshold (>0.25 or <0.25) without any intervention. Although underpowered, those at the extremes of the score showed consistency over time. We also explored the potential for modifiability in the supplementary data using external datasets (Supplementary Figure).

As a conceptual hypothetical framework, we calculated the PRIMUS signature as a retrospective example using an external database in a non-PCa cohort. This was derived from an external study of patients with IBD using the data from Human Microbiome Project 2 and Crothers et al’s [17] study. We aimed to determine whether the PRIMUS score could be calculated from another cohort and whether the microbiome scores could hypothetically be modified. When fecal microbiota transplantation was performed in these unadjusted cohorts, the PRIMUS signature appeared to be higher in men with IBD. In an interventional clinical trial of fecal transplant for IBD, the calculated PRIMUS signature was reduced with fecal microbiota transplantation, becoming more similar to the donor stool, suggesting potential for modification (Supplementary Fig. 13).

This study has several limitations. We used two patient groups with notable demographic differences that may have introduced an unaccounted bias. Additionally, we relied on 16S short-read sequencing, which offered less depth of DNA coverage than shotgun approaches; nevertheless, 16S is less expensive and has large comparator databases. Prior to recruitment, we were unaware whether patients with “not reported” in their family history, likely due to limited data availability in the record for the biopsy cohort, have a higher risk of PCa. Second, different collection methods, namely, stool, rectal swabs, and glove tip collections, were used; however, we have previously shown that these methods do not substantially affect the microbiome analysis findings [18]. Therefore, it is possible that skin/perianal microbes could be overemphasized in the score due to the collection technique. Our analysis may lead to overfitting as we used the Linear discriminant analysis Effect Size method to select candidates before artificial intelligence methods as well as cross-validation, both of which could lead to overfitting of the data. Finally, while other groups have reported on the gut microbiome in the context of outcomes related to PCa treatment, the current study remains the largest longitudinal assessment on the association between the gut microbiome and PCa incidence [19]. Our dataset establishes the possibility of a modifiable risk factor, and a prospective interventional trial is warranted to determine modifiability. Our results may not be generalizable to other populations. All risks and biases of the study were inherent to the study design. We anticipate further clinical validation of the PRIMUS biomarker by expanding the population to include broader geographic locations, as well as various disease stages (such as incident, active surveillance, and advanced), with incorporation into interventional clinical trials.

## 5. Conclusions

Our key findings suggest that a gut microbiome signature may provide additional insights into PCa risk and could be considered a component of lifestyle assessment. The implications of this research could lead to microbiome factors serving as a potential tool in evaluating the current and future PCa risk.

## Supplementary Material

1

## Figures and Tables

**Fig. 1 –:**
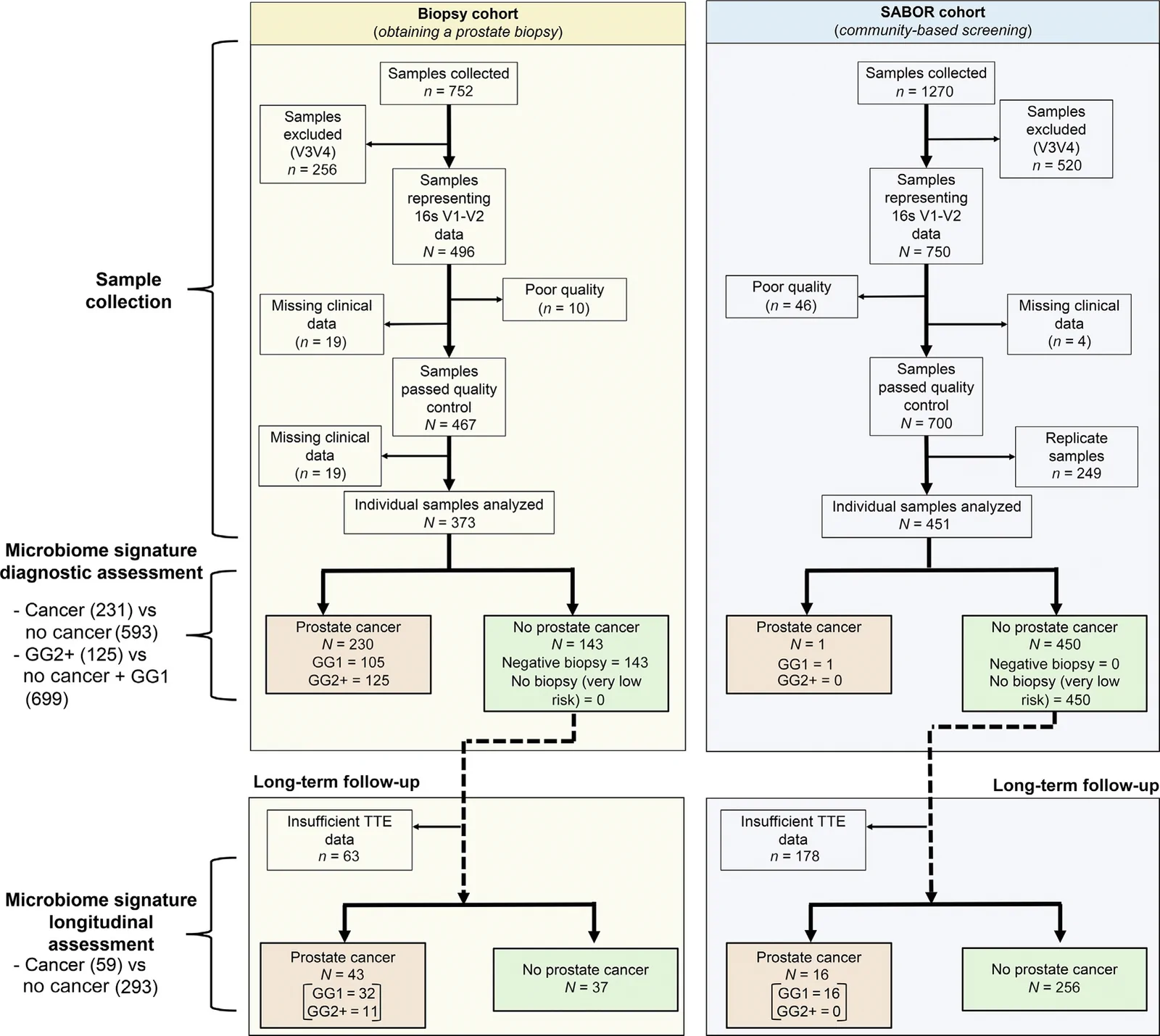
Flow diagram. The flow diagram describes the groups and number of specimens obtained. GG1 = grade group 1; GG2+ = grade group >2 prostate cancer; PCa Dx = prostate cancer diagnosis; SABOR = San Antonio Biomarkers of Risk; TTE = time to event.

**Fig. 2 –:**
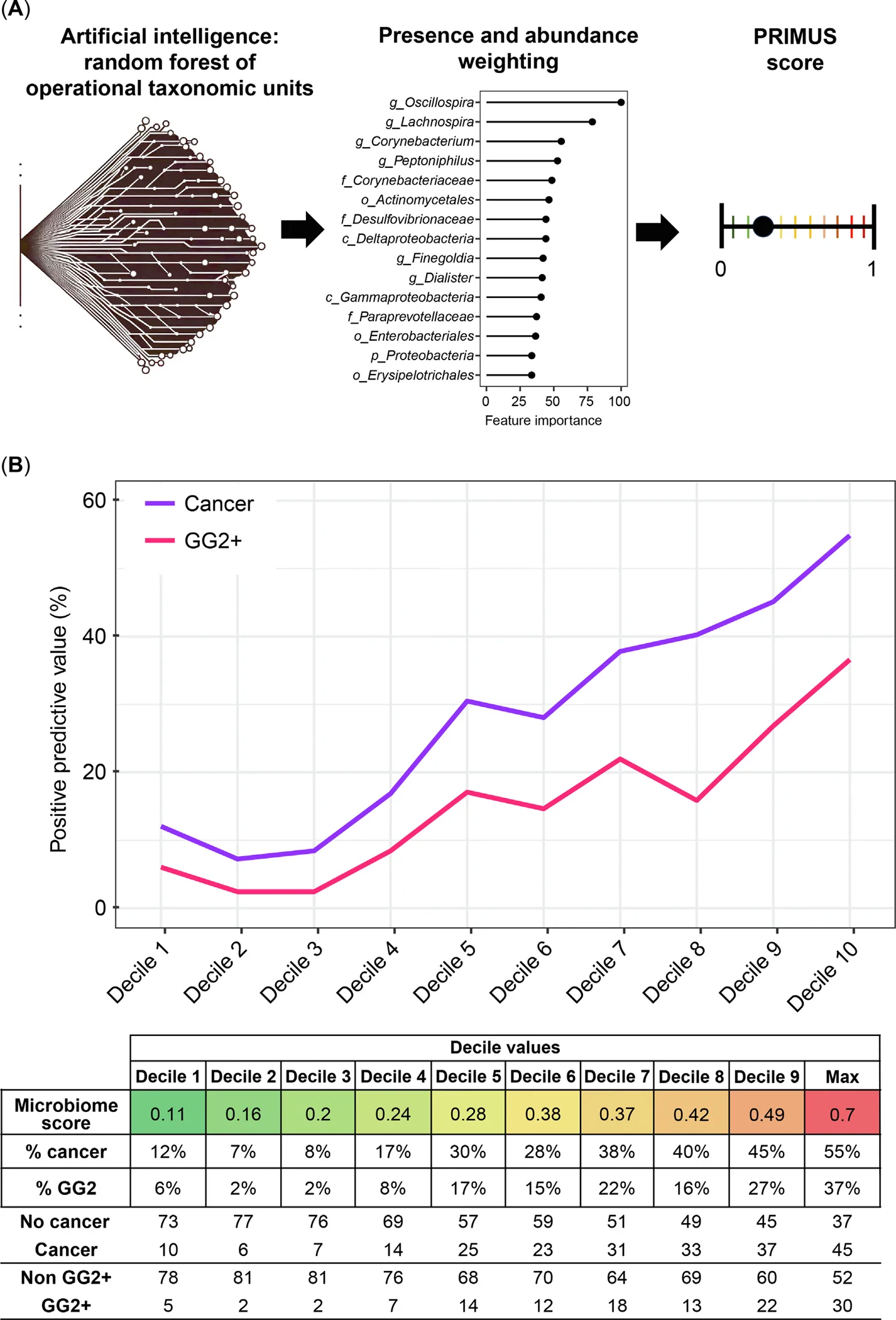
PRIMUS signature creation and biological gradient. (A) PRIMUS signature. After microbial DNA isolation, sequencing, and operational taxonomic unit identification, we performed a random forest analysis to determine the combination of microbes associated with prostate cancer. (B) Line graph showing the biological gradient of PRIMUS. We divided the PRIMUS signature into deciles using a color-coded bar graph to demonstrate the representative score. The chart shows the proportions of prostate cancer (% cancer) and GG2+ prostate cancer (% GG2). The proportions are also represented in the line graph, showing the increasing cancer risk with a rising PRIMUS signature. The final two rows of the table show the raw number of patients constituting the proportions. GG2 + = grade group >2 prostate cancer; PRIMUS = Prostate Cancer Risk Insight using Microbiome UnderStanding.

**Fig. 3 –:**
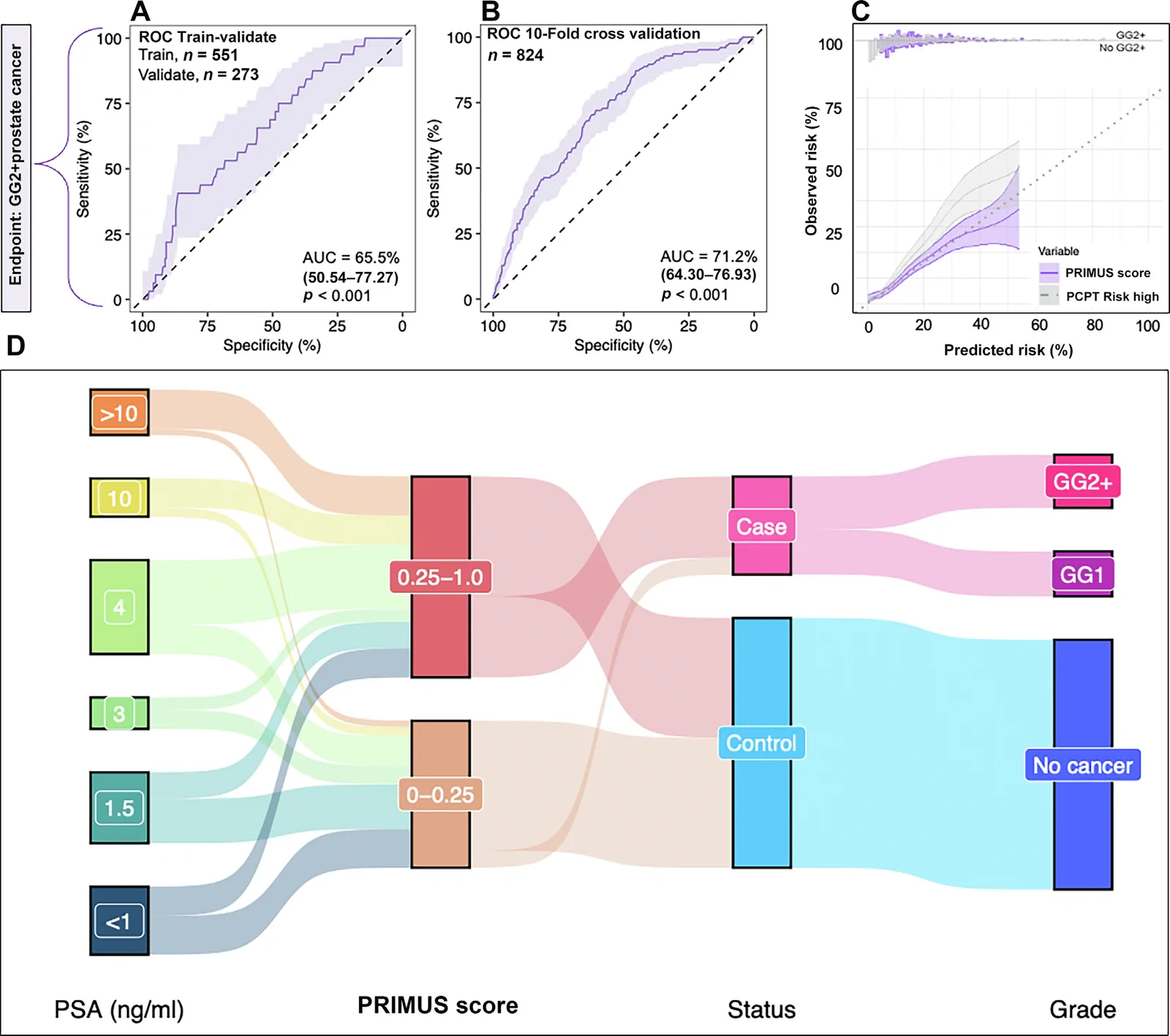
Prostate cancer detection and performance. Operational performance of microbiome score. The ROC-AUC curve depicts the (A) training (two-thirds, *n* = 551) and validation (one-third *n* = 273) model development with associated histograms for cancer and no cancer versus. GG2+. (B) Operating characteristic curve of GG2+ using a ten-fold cross-validation method. (C) Calibration curve of PRIMUS and PCPT high-risk PCa%. The top of the figure shows a histogram noting the distribution of PRIMUS signatures and PCPT risk and whether they were observed as having cancer (top) or not having cancer (bottom). (D) Alluvial plot showing microbiome scores by PSA level cutoff. The 0.25 PRIMUS signature cutoff depicts positive or negative test results, followed by “case” (cancer) or “control” (no cancer) categories. AUC = area under the curve; GG2+ = clinically significant prostate cancer to include grade group ≥2; PCa = prostate cancer; PCPT = Prostate Cancer Prevention Trial; PRIMUS = Prostate Cancer Risk Insight using Microbiome UnderStanding; PSA = prostate specific antigen; ROC = receiver operating characteristic.

**Fig. 4 –:**
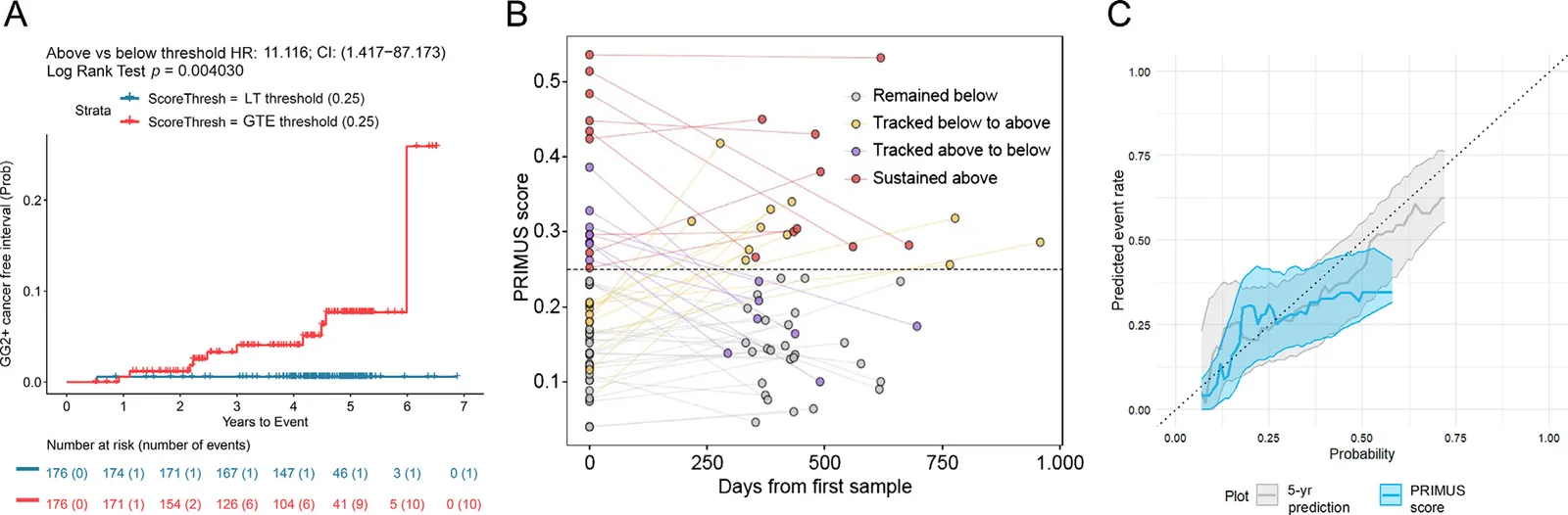
Longitudinal time-to-event cumulative risk analysis. (A) Time to GG2+ PCa diagnosis. A Kaplan-Meier plot shows decreased GG2+ PCa-free survival in patients with a baseline microbiome score of ≥0.25. (B) Line chart showing changes in the microbiome over time. The *x*-axis shows the PRIMUS signature, and the *y*-axis shows the time in days. Fifty-three patients without cancer were recruited on day 0, and the data for one or more downstream time points were plotted with colored dots. The line represents the changes over time (median of 1.17 yr, interquartile range: 0.99–1.34 yr). The gray dots and lines represent scores that started below the threshold of 0.25 (*n* = 25, 47.2%), the yellow dots/lines represent scores that started below the threshold and were identified as above the threshold at a later date (*n* = 12, 22.6%), the purple dots/lines represent scores that started above the threshold and were identified as below the threshold (*n* = 7, 13.2%), and the red dots/lines represent scores that started above and remained above the threshold (*n* = 9, 17.0%). None of the patients developed PCa during the follow-up period, although six patients underwent a prostate biopsy. The longitudinal cohort was diverse, with 30% (*n* = 16) of the patients being of African descent, 38% (*n* = 20) being Latino/Hispanic, and 32% (*n* = 17) being of European descent. (C) Calibration curve comparing PRIMUS signatures with a 5-yr PCa prediction model. The dotted line represents perfect prediction. CI = confidence interval; GG2+ = clinically significant prostate cancer to include grade group ≥2; GTE = greater than or equal to; HR = hazard ratio; LT = less than; PCa = prostate cancer; PRIMUS = Prostate Cancer Risk Insight using Microbiome UnderStanding; Prob = probability; ScoreThresh = score threshold.

**Table 1 -:** Characteristics of the study population grouped by cancer grade

	GG2+ (*N* = 125)	GG1 (*N* = 106)	Negative biopsy (*N* = 143)	Low risk (*N* = 450)

Age (yr)	68.0 (37.0–81.0)	66.0 (47.0–79.0)	66.0 (37.0–81.0)	68.5 (40.0–87.0)
Body mass index	28.8 (19.3–46.2)	29.0 (18.7–45.9)	29.1 (18.7–47.8)	27.6 (14.6–71.2)
DRE, *n* (%)				
Normal	64 (51.2)	79 (74.5)	102 (71.3)	404 (89.8)
Abnormal	57 (45.6)	22 (20.8)	19 (13.3)	34 (7.6)
Not performed	4 (3.2)	5 (4.7)	22 (15.4)	12 (2.7)
Race, *n* (%)				
White	74 (59.2)	54 (50.9)	74 (51.7)	395 (87.8)
Black	38 (30.4)	35 (33.0)	50 (35.0)	50 (11.1)
Other	13 (10.4)	17 (16.0)	19 (13.3)	5 (1.1)
Ethnicity, *n* (%)				
Non-Hispanic	89 (71.2)	83 (78.3)	108 (75.5)	309 (68.7)
Hispanic	34 (27.2)	20 (18.9)	30 (21.0)	141 (31.3)
Other	2 (1.6)	3 (2.8)	5 (3.5)	0 (0)
Family history of PCa, *n* (%)				
No	106 (84.8)	83 (78.3)	121 (84.6)	380 (84.4)
Yes	3 (2.4)	5 (4.7)	4 (2.8)	69 (15.3)
Not reported	16 (12.8)	18 (17.0)	18 (12.6)	1 (0.2)
Prostate-specific antigen level (ng/ml)	8.06 (0.140–295)	5.89 (0.270–27.6)	6.33 (0.800–19.3)	1.4 (0.100–54.9)
Observed microbial species	62 (29.0–85.0)	63 (43.0–84.0)	63 (34.0–94.0)	63 (38.0–158)
Shannon diversity	3.56 (1.21–4.43)	3.61 (0.952–4.39)	3.54 (1.12–4.39)	3.54 (1.90–4.59)
PRIMUS signature	0.376 (0.07–0.70)	0.365 (0.05–0.67)	0.364 (0.06–0.66)	0.216 (0.02–0.69)

DRE = digital rectal prostate examination; GG1 = prostate cancer to include grade group 1; GG2+ = clinically significant prostate cancer to include grade group ≥2; PCa = prostate cancer; PRIMUS = Prostate Cancer Risk Insight using Microbiome UnderStanding.

## Data Availability

Data are available for bona fide researchers who request it from the authors.

## ADVANCING PRACTICE

### What does this study add?

Our investigation is the largest to examine the association between gut bacteria and prostate cancer using longitudinal follow-up data prior to diagnosis. It enhances the growing understanding of the role of lifestyle factors in prostate cancer risk. We suggest that a gut microbiome signature linked to prostate cancer could be a potential tool for use in future clinical trials.

### Clinical Relevance

This study identifies a gut microbiome–based signature (PRIMUS) that is associated with prostate cancer risk, offering an objective measure of lifestyle-related risk. While not intended to guide biopsy decisions, this tool could enable personalized risk stratification and support future preventive strategies targeting modifiable microbiome factors. Associate Editor: Derya Tilki.

### Patient Summary

We assessed men undergoing prostate cancer screening. We found that the bacterial microbes in the feces of men with a higher risk of developing prostate cancer are different from those of men who have a lower risk. Using these data, we developed a test that can be used in the assessment of prostate cancer lifestyle risk.

## Appendix A. Supplementary data

Supplementary data to this article can be found online at https://doi.org/10.1016/j.euf.2026.01.001.
