# Supplementary material for: Gut Microbiome as a Lifestyle Risk Factor Associated with Prostate Cancer
Source: Eur Urol Focus. Author manuscript; Available in PMC 2026 Oct 1. (PMC13630277; doi:10.1016/j.euf.2026.01.001)
Supplement: Supplementary file 1 [file NIHMS2201134-supplement-1.docx]

**Supplementary materials**

**1. Supplementary Methods**

**2. Supplementary Results**

**3. Supplementary Tables**

**4. Supplementary Figures**

**5. Supplementary References**

**1.	Supplementary Methods**

## Inclusion, exclusion, and follow-up

### San Antonio Biomarkers of Risk (SABOR) cohort

One group included men undergoing prostate cancer (PCa) screening as part of the SABOR cohort (IRB#: HSC20000030H), an 18-year prospective cohort study comprising 4,174 participants undergoing regular serum prostate specific antigen (PSA) testing as part of a clinical validation at an institutional site for the National Cancer Institute’s Early Detection Research Network. [1] The SABOR cohort comprised a patient population at usual risk who underwent PCa screening within the community and presented annually at UT Health San Antonio outpatient clinics for PCa screening. At the SABOR visit, they were recruited to contribute a fecal sample via swab or glove tip after a digital rectal examination for microbiome research purposes. Therefore, these men were not expected to undergo biopsy unless indicated by a community physician and were at a low risk for PCa. Patients visited our SABOR-dedicated clinics for annual screening and follow-up. These men (SABOR cohort) consented to the rectal swab collection procedure; all data were collected prospectively. We performed an annual follow-up at that visit, and in March 2023, we conducted a follow-up telephone call to confirm that all data were captured and updated. Patients with PCa were not included in the study, and if they had cancer at follow-up, their cancer status was documented, and they were no longer included. Those who did not respond after three contacts were deemed to be “lost to follow-up” and were censored at the date of the last contact in longitudinal analysis.

### Pre-biopsy cohort

## The second group comprised men whose data were part of a genitourinary repository (IRB#: HSC20050234H) and who decided to undergo prostate biopsy. Biopsy was the requested inclusion criterion and no exclusion criteria were applied. Prior to the biopsy, we obtained a rectal culture at the time of scheduling for prostate biopsy as part of the standard-of-care antibiotic selection strategy for infection prevention at the San Antonio Audie L. Murphy VA hospital outpatient urology clinic. The patients were not receiving antibiotic therapy at the time of collection, which is typically performed on the morning of the biopsy. During the collection of this standard care swab, two swabs were obtained and placed in a BBLTM CultureSwabTM Collection and Transport System (BD, Franklin Lakes, NJ, USA). Therefore, the additional swab was saved instead of being disposed of, was de-identified, and sent to the genitourinary biospecimen bank for bacterial DNA isolation. The requirement for consent was waived for this cohort, as the specimens were collected for the repository without risk to the patient; specimens were de-identified, and an independent, honest broker was used for data collection. For follow-up, the honest broker performed a chart review and de-identified it before sharing it with the study team. The date of the last note in the VA electronic medical record was considered the date of the last contact in longitudinal analysis.

### **Sample collection, preparation, and DNA isolation**

*Sample collection and preparation*

All specimens in the biopsy cohort were prospectively collected before the antibiotic therapy. We used both rectal swabs and glove tips for fecal microbiome collection, and a previous study found that these two methods were highly correlated in terms of DNA yield and gut microbiome composition. [2] The provider performed the rectal swab technique (as previously referenced), used sterile Medline E-Z Lubricating Jelly, placed the swab approximately 2 inches into the rectum, and then turned the swab 360°. Once the collection was completed, the rectal swab was removed, and the samples were examined for stool. If no fecal material was collected, the swab was re-inserted, and the process was repeated. The swab was placed in a 15-mL sterile centrifuge tube containing 1 mL of phosphate-buffered saline (PBS). For patients in the SABOR group, a glove tip was used after a digital rectal examination. The sample collector wore two sets of gloves to avoid specimen contamination, and the index finger of the outer glove was used for specimen collection. The glove was then removed, and the specimen was sent for processing. Swab specimens were stored at 4 °C following collection and were transported to the laboratory within 4 hours of collection. Swabs and fecal material were stored in PBS at -20 °C until DNA isolation was performed.

*DNA isolation and sequencing*

We obtained a visible amount of stool from glove tips and swabs for sample input. DNA was isolated from fecal samples using a standard operating procedure. [3] We purified genomic DNA from the respective fecal samples using the QIAamp® Fast DNA Stool Mini Kit according to the kit protocol (Qiagen, Germantown, MD). We sequenced 16S rRNA (V1–V2) using primers, 27F (forward primer: 5ʹ- AGA GTT TGA TCM TGG CTC AG-3ʹ) and 355R (reverse primer: 5ʹ– GCT GCC TCC CGT AGG AGT-3ʹ) with an Illumina overhang primer (forward overhang primer sequence: 5ʹ-TCGTCGGCAGCGTCAGATGTGTATAAGAGACAG-3ʹ + forward 27F; reverse overhang primer sequence: 5ʹ-GTCTCGTGGGCTCGGAGATGTGTATAAGAGACAG-3ʹ + reverse 355R [27F/355R]). [4] The DNA concentration was measured using a Thermo Scientific NanoDrop instrument (Waltham, MA, USA). We used the 16S V3–V4 variable region following the Illumina 16S metagenomic library preparation guide for robustness. [4] The final libraries were quantified, normalized, pooled, and sequenced via paired-end sequencing (2 × 300 bp) using the Illumina MiSeq platform (San Diego, CA, USA). An average of 264,727 raw paired reads per sample was generated, with read lengths of 2 × 150 bp. Sequences were exported as FASTQ files.

*PCa microbial pathways and disease states*

Normalized taxonomic abundance profiles per sample were analyzed for functional inference using Phylogenetic Investigation of Communities by Reconstruction of Unobserved States (PICRUSt). The resulting 328 quantified functional features were compared between groups using the Mann–Whitney U test with false discovery rate P value adjustment to identify the statistically significant differences.

*Assessment of polyketide synthase gene*

We used the previously isolated DNA to perform polymerase chain reaction (PCR) sequencing with primers for colibactin N (*clbN*) (Forward: 5ʹ-GTTTTGCTCGCCAGATAGTCATTC-3ʹ, Reverse: 5ʹ-CAGTTCGGGTATGTGTGGAAGG-3ʹ) and colibactin B (*clbB*) (Forward: 5ʹ-GATTTGGATACTGGCGATAACCG-3ʹ, Reverse: 5ʹ-CCATTTCCCGTTTGAGCACAC-3ʹ). The amplicon sizes were 733 bp for *clbN* and 579 bp for *clbB*. Primers were chosen based on previous studies. [5, 6] The patient was classified as polyketide synthase (PKS) ^+^ and capable of producing colibactin if either *clbN* or *clbB* tested positive.

*Assessment of suspected causal organisms*

We retrieved taxonomic associations from two Mendelian randomization studies and mapped the reported taxa to the closest representative taxa in our dataset. [7, 8] We collected and analyzed the relative abundance values of the selected representative taxa using hierarchical clustering with a Euclidean distance metric (R package *pheatmap* v1.0.12) and dimensionality reduction with t-SNE (R packages *Rtsne* v0.15 and *UMAP* v0.2.8). Prior to t-SNE, a small amount of random noise was added to each value (Gaussian distribution with mean 0 and standard deviation 0.0001). The t-SNE analysis used a "perplexity" setting of 5, while UMAP was run with default settings.

## PRIMUS score development

### Data preparation

Raw paired-end 16S rRNA reads were merged into consensus fragments using fast length adjustment of short reads and filtered for quality and length using Trimmomatic (non-default parameters SLIDINGWINDOW:25:20 MINLEN:200) and QIIME (v1.8.0) (split_libraries_fastq.py with non-default parameters -n 1 -q 19 --max_bad_run_length=5 --min_per_read_length_fraction=0.1). [9–12] The passing sequences were trimmed using primers, evaluated for chimeras, and screened for human-associated contaminants using Bowtie2 (parameters --end-to-end --sensitive -k 1) against the human reference genome GRCh37, followed by a broad BLASTN (--disable_low_complexity_filter --e_value 1e-4 --word_size 16) search against the Greengenes 16S rRNA database (v13.5) to confirm 16S rRNA similarity and generate a high-quality *16S* sequence set. [13]

### Taxonomic assignment

Using default parameters, the primary taxonomic assignment was performed using closed-reference operational taxonomic unit (out) selection against the Greengenes database (v13.5). Chloroplast- and low-frequency reagent-associated contaminant taxa were excluded (i.e. those matching c__Chloroplast, c__Alphaproteobacteria, f__Propionibacteriaceae, o__Oceanospirillales, f__Intrasporangiaceae, f__Micrococcaceae, f__Cytophagaceae, o__Hydrogenophilales, f__Alcaligenaceae, f__Burkholderiaceae, f__Burkholderiales_incertae_sedis, f__Comamonadaceae, or f__Oxalobacteraceae). Downstream contaminant-filtered taxonomic profiles were rarefied to 15,000 sequences per sample for comparative analysis. Computational analyses were performed separately for V1–V2 and V3–V4 amplicons.

### Calculation of diversity scores

Alpha diversity calculations emphasizing richness and evenness (observed species, Shannon diversity) were performed using in-house Perl code. Beta diversity comparative analysis (Principal Coordinates Analysis using Bray–Curtis) was performed using the *vegan* R package (v2.5) for PERMANOVA calculations. [14]

### Determining relative cancer association

We assessed the differential abundance between patient groups of interest using the non-parametric Mann–Whitney U test with false discovery rate-adjusted p-values. [15, 16] Linear discriminant analysis (LEfSE using default parameters) was performed using the Galaxy server to compare PCa with no cancer and grade group (GG) 2+ with no cancer. [17, 18] LDA was used to identify key microbial differences.

## PRIMUS score robustness

Our initial sequencing data included the variable region of the 16S rDNA gene V1–V2. Subsequently, we sequenced the V3–V4 and V1–V2 regions to evaluate the relationship between PRIMUS scores from the two regions. For samples from each region, the percentage abundance values of each input taxon were generated and subjected to the PRIMUS model. Correlation analysis (Pearson method) was performed between the PRIMUS score calculated using the V1–V2 profiles and the PRIMUS score calculated using the V3–V4 profiles with the resultant *r* value.

### **PRIMUS score and threshold selection**

Statistical univariate analysis of demographic data included the chi-squared or Fisher’s exact test for categorical variables and the Student’s t-test for continuous variables. We selected the optimal threshold for analysis using the area under the curve (AUC). Logistic regression was used for multivariate comparisons to determine whether the microbiome score was significant in predicting the dichotomous outcome of clinically significant PCa (csPCa) while controlling for other factors, including serum PSA levels. The cut-off threshold of 0.25 was determined by maximizing the average sensitivity and specificity on the AUC curve while maintaining a minimum sensitivity of 80%. We tested this approach using the Youden index, which represents the maximum cut-off value (Supplementary Figure 5). [20]

## **PRIMUS comparisons to PSA with decision-curve analysis (DCA)**

We examined the clinical benefits of microbiome predictors for csPCa and any grade of PCa using DCA implemented in the R package *dcurves* (v0.4). [21] The accuracy of the microbiome score and Prostate Cancer Prevention Trial (PCPT) risk calculator in predicting low-risk and high-risk (GG2+) PCa was evaluated using the AUC, prediction calibration, and DCA. The calibration compares the predicted risk to the observed risk and was estimated using the *probably* R package with the function *cal_plot_logistic.* DCA provides the clinical net benefit of using the predicted risk as a decision rule for treatment (biopsy) over a range of treatment probability thresholds. The net benefit (NB) is equal to the quantity *NB = sensitivity × prevalence – (1 – specificity) × (1 – prevalence) × w,* where *w* is the odds at the threshold probability. The decision curves were estimated using the *dcurves* R package with the *dca* function. [21] The incremental benefit of the microbiome score was evaluated using logistic regression.

## **PRIMUS comparisons to PCPT 2.0**

PRIMUS and the corresponding PCPT risk scores per participant were transformed to percentile values and evaluated in the context of observed PCa and high-grade PCa risk. Observed risk was calculated as the percentage of PCa (or high-grade PCa) positive participants in contiguous windows of the 0.05 percentile. Locally Estimated Scatterplot Smoothing model fitting with 95% confidence interval (CI) estimation was performed for each risk score and observed risk measure.

## Longitudinal analysis for predictive analysis

When generating the PRIMUS score, we assumed that using the same dataset would induce overfitting of the PCa outcome. Therefore, we followed up with patients without a cancer diagnosis over time to test the predictive ability of the generated score. We tested the ability of the PRIMUS score at the optimum threshold determined from the AUC curve to stratify men from the entire cohort into high- or low-risk categories. We excluded men diagnosed with PCa within 6 months of microbiome collection to ensure that this was a true longitudinal cohort, with a group assumed not to have PCa at the outset. If an individual was biopsied within 6 months of swab collection, the assumption was that a biopsy was obtained because of a high suspicion of PCa. This analysis aimed to determine the predictive value of the baseline PRIMUS score. We then used a Kaplan–Meier curve for visualization and the log-rank test to determine the occurrence of a significant difference in the incidence of PCa or csPCA. In the biopsy cohort, the medical records were used for follow-up, noting any additional biopsies and the date of the last contact, and the patient was censored after that date. For the SABOR cohort, annual phone calls or visits were a standard part of the study. A new diagnosis of PCa was documented, and the patient was lost to follow-up when the last contact was used. We assessed the accuracy of the microbiome score for predicting the 5-year PCa risk using a calibration curve in the cohort comprising the screening group and the biopsy-negative follow-up group. The microbiome score was the only predictor in the Cox proportional hazards model used to create a 5-year PCa risk prediction. We calculated the calibration curve (observed vs. predicted risk) using inverse probability weights for censored observations implemented in the pec R package *calplot* function and estimated 95% CIs using 1000 bootstrap resamples. The microbiome score was compared with the published 5-year predictor from Gelfond et al. [22] We used the concordance index (c-index) to evaluate the performance of the PRIMUS score, accounting for clinical variables in a survival-prediction model to measure the probability that an individual observed to have an earlier event had a higher predicted risk (expected in observed cases). We used a previously published 5-year PCa prediction model to account for clinical variables. [22]

## PRIMUS score confirmation of elevation in inflammatory bowel disease (IBD)

First, we obtained a publicly available 16S rRNA gene sequence data subset from the Human Microbiome Project 2 (HMP2) of patients with IBD. We then calculated the PCa-specific microbiome score for patients with PCa in this integrative human microbiome project sub-analysis of IBD. [23] We obtained publicly available data from https://www.hmpdacc.org/ihmp/, which contained the V4 region sequence (MiSeq platform [Illumina] using 2 × 250 bp paired-end reads), which were analyzed using the same bioinformatics methodology as our cohort, resulting in normalized taxonomic profiles that were submitted to our pre-trained machine learning model for PRIMUS scoring. We analyzed the PRIMUS scores of patients with and without IBD and divided them into male and female groups. The purpose of calculating the score for women was to determine whether the PRIMUS score was unique to men. We compared the groups using the Mann–Whitney U test and visualized the microbiome score using box and whisker plots.

## External data sources

### HMP2

HMP2 (https://www.hmpdacc.org/ihmp/) was an observational study of 90 individuals whose stool microbiome was profiled for 1 year. The cohort was specifically selected due to the patient’s diagnosis of IBD proven by colonoscopy. We have shown a correlation between our two 16s sequencing variable regions (V1,V2 and V3,V4). The HMP2 dataset used 16S rDNA V4 region amplification and was sequenced on the same platform as our samples (MiSeq platform, Illumina) using a similar depth of 2×250 bp paired-end protocol targeting at least 10,000 reads per sample.

### NCBI’s SRA Database Repository

To test if the PRIMUS score could be modified, we identified a fecal microbiota transplant (FMT) study (BioProject PRJNA475599) that used a similar 16s protocol. The DNA extraction used the MoBio Powersoil 96 kit and 16S of the V4 region. Twelve patients with active ulcerative colitis were randomized in a 1:1 ratio to receive FMT induction by colonoscopy, followed by 12 weeks of daily oral administration of frozen encapsulated cFMT or sham therapy. The individuals were followed up for 36 weeks. Ribosomal 16S rRNA sequencing was used to assess donor-induced changes in the gut microbiota.

## Mendelian randomization and our dataset

We assembled taxonomic categories associated with an increased or decreased risk of PCa from two Mendelian randomization studies. [24, 25] We then collected these taxonomic abundance values per sample in our dataset and performed unsupervised hierarchical clustering, t-distributed stochastic neighbor embedding (t-SNE), Uniform Manifold Approximation and Projection (UMAP) t-SNE, and UMAP dimensionality reductions to evaluate whether clusters of cancer or non-cancer participants were present.

**2.	Supplementary Results**

## Ribosomal 16S variable region selection

During this study, the V4 variable region became more favored in microbiome studies. To avoid losing all V1–V2 data, we started sequencing the V3–V4 data along with our V1–V2 sequencing runs. We then compared the PRIMUS score using V1–V2 data with the PRIMUS score using V2–V4 data. We plotted the data on a correlation plot and noted a significant correlation between the microbiome scores of the two regions (Pearson’s correlation coefficient= 0.77, *P*<0.001; Supplementary Figure 4).

## Alpha and beta diversities

The overall diversity profiles in the four groups were as follows: GG2+, GG1 (Gleason +3), negative biopsy, and low risk of cancer (such as men in screening with a sufficiently low risk of PCa who did not need a biopsy; Table 1). Alpha diversity did not differ significantly between the groups (Supplementary Figure 1), and subsequent subgroup analyses of cancer status and race revealed no significant differences (all *P*>0.05). However, we observed differences in beta diversity comparisons according to cancer status (*P*<0.01). We also investigated differences among race and ethnicity. Although the overall noted principal coordinates analysis plots had significant overlap, beta diversity was statistically different between the groups (*P*<0.01; Supplementary Figure 2)**.** Although alpha and beta diversities are generic forms used to compare microbiomes, we further assessed specific bacteria as OTU and imputed their potential functions.

## PRIMUS score machine learning

After sequencing, quality control, and metadata hygiene, we first identified the bacteria associated with PCa using linear discriminant analysis (LDA). In the LDA, a cut-off of >2.4 was used to separate valuable microbial profiles and thus determine whether the microbes are more or less likely to be associated with PCa (Supplementary Figure 3). We identified 39 OTUs with microbial representatives across families, orders, and species. *Proteobacteria, Gammaproteobacteria*, and *Enterobacterales* were more likely to be identified in men with PCa, whereas *Bacteroidetes, Ruminococcaceae,* and *Oscillospira* were more likely to be highly abundant in men without cancer. However, to ensure that each bacterial abundance and its impact on the association with PCa were accounted for, we used the random forest machine learning model to apply a selection criterion to assess the development of a microbiome score associated with PCa. The microbiome score (output) was represented by a number ranging from 0 to 1, with an increasing number of genes associated with PCa. We showed the top 15 microbes and their feature importance on the PRIMUS score, noting that *Oscillospira* had the greatest influence on the PRIMUS score, having the largest feature importance.

## Threshold selection of PRIMUS score

The optimal threshold for the PRIMUS score was 0.25, which was determined using the Youden index and maximizing the average sensitivity and specificity of the AUC. The sensitivity, specificity, positive predictive value (PPV), and negative predictive value (NPV) at this threshold were 82.7%, 52.1%, 40.2%, and 88.5%, respectively. Owing to the recent emphasis on the detection of clinically significant PCa, we also examined the ability of the PRIMUS score to predict csPCa, which is defined as grade group 2 or higher (i.e., GG2+). The receiver operating characteristic (ROC) curve had an AUC of 0.712 (95% CI 0.643–0.769). The threshold used to establish a positive test was the same, leading to sensitivity, specificity, PPV, and NPV of 84.8%, 46.9%, 22.2%, and 94.5%, respectively.

We then performed a 10-fold cross-validation of this cohort and generated the AUC (Figure 3). Cross-validation (n=824) usually outperforms other validation techniques but has a higher risk of overfitting; therefore, we used traditional train-test validation sets with the same data to improve robustness. We divided the cohort into training (2/3, n=551), created a new microbiome score, and tested the score in the remaining cohort (1/3, n = 273). We then compared the generated scores using a random forest to confirm the similarity between the two model training sets and identified a significant positive correlation between the two approaches (Pearson, r=0.79; *P*< 0.001; Figure 3)*.* We developed a PRIMUS score to detect PCa, including patients with low-risk PCa, compared with patients without cancer. Using a training/validation set, the microbiome score achieved an AUC of 0.686 (95% CI, 0.681–0.687, *P*<0.001). In the second analysis, using a 10-fold cross-validation approach, the microbiome score had an AUC of 0.717 (95% CI 0.713–0.717, *P*<0.001).

## Previous Biopsy and PRIMUS score

Men who previously underwent biopsy and were negative have a lower risk of prostate cancer and subsequent biopsy due to benign prostate hyperplasia driving higher PSA, but they also may have been exposed to prophylactic antibiotics (typically fluoroquinolones). A concern in this population is that antibiotics could have inherently altered the microbiome. We did not perform a full antibiotioc analysis, but we could compare previous biopsy to no previous biopsy. Therefore,we compared the PRIMUS scores of men with and without PCa in the groups that did and did not have a prior prostate biopsy. We noted differences only in cancer versus no cancer, and no differences were observed between patients with and without a previous biopsy (Supplementary Figure 7).

## PRIMUS score and IBD

As a hypothetical example to test the hypothesis of potential modifiability, we obtained 16S microbial sequencing data from a sub-study of the integrative human microbiome study on IBD. We selected IBD owing to its associated future risk of PCa and determined whether the PRIMUS score was unique to men. We observed significantly higher PRIMUS scores among patients with PCa in men with IBD than in controls (Figure 5, *P*=0.0025), but the scores were not different between women with or without IBD. Therefore, the PRIMUS score as a PCa risk assessment tool is unique to men, and bacteria may have hormonal implications. Second, we obtained external *16S* microbiome data from an interventional clinical trial to improve IBD with fecal transplantation [26]. We obtained these data to determine whether the PRIMUS score could be reduced (modified) in men with high PRIMUS scores. After obtaining publicly available data, we calculated the PRIMUS score of the donor stool samples. We found it to be significantly lower than that in baseline stool samples from patients with IBD (Figure 5, *P*<0.001). Based on these findings, we compared the PRIMUS scores of men who received fecal microbiota transplantation (intervention group) and those who did not (placebo group) 25–35 days after FMT. The microbiome score of the intervention group was low and similar to that of the donors (*P*=0.12), whereas that of the placebo group was significantly higher than that of the intervention group (*P*=0.002). Thus, an external validation of patients with IBD known to have gut dysbiosis and are at risk for PCa showed higher PRIMUS scores, which were unique to men and could be modified by FMT. The population of this cohort differs, and they have a distinct disease process; however, the overall example serves as proof of concept that the microbiome may be modifiable.

## Microbiome-disease states and pathways

PICRUSt was used to determine pathways or disease states related to the PRIMUS score and verify associations (Supplementary Figure 8) [15]. We identified the top three associations among the colon, small cell lung, and PKS. To verify the colon and lung cancer (not specifically small cell) associations, we utilized external cohorts from previous publications; however, we were unable to show differences in PRIMUS scores between those with and without cancer stratified by sex (males, *P*=0.19 and *P*=1.0, respectively). [27, 28] We performed a subsequent analysis to examine the findings in our longitudinal cohort, noting whether PKS^+^ would provide additional benefits when added to the PRIMUS score. The addition of PKS^+^ did not change the AUC for cancer prediction beyond the PRIMUS score (all AUCs did not change). Those with a high PRIMUS score and PKS^+^ were at the highest risk of a future diagnosis of cancer or GG2+ cancer; however, this was not statistically different to stratify those with low PRIMUS PKS^-^ vs. PKS^+^ (HR 0.55, 95% CI 0.14–2.21, *P*=0.39) or with high PRIMUS PKS^+^ vs. PKS^-^ (HR 1.46, 95% CI 0.75–2.84, *P*=0.26; Supplementary Figure 9).

## Mendelian randomization and our dataset

Overall, we could not confirm that the composition of the selected taxa from previous publications was associated with PCa status in our data (Supplementary Figure 10).

## PRIMUS score as a modifiable biomarker

We obtained publicly available microbial sequence data from a clinical trial by Crothers et al. using fecal microbiota transplant in patients with IBD [18]. The study used a single-center, prospective, 1:1 randomized pilot study using daily oral FMT for 36 weeks. This study involved *16S* rRNA gene V4 sequencing of samples from two donors and 12 participants (six with interventions and six as controls). The two donors were selected because of their high (top quintile, 11.11 mmol/g, and 12.67 mmol/g stool) levels of butyrate production. We calculated the PRIMUS microbiome scores of donors in both the intervention and control groups at each time point. We used the unpaired Mann–Whitney U test to compare microbiome scores before and after the intervention, as well as Loess model fitting, examining the average changes in scores among treated and placebo groups over time.

**3.	Supplementary Tables**

**Supplementary Table 1**. Demographics of the two study population cohorts

|  | **Biopsy Cohort** | **Screening Cohort** | **P-value** |
| --- | --- | --- | --- |
|  | (n=373) | (n=451) |  |
| **Age** | 67.0 [37.0–81.0] | 69.0 [40.0–87.0] | <0.001 |
| **Body mass index** | 29.0 [18.7–47.8] | 27.6 [14.6–71.2] | <0.001 |
| **Digital rectal examination** |  |  | <0.001 |
| Normal | 244 (65.4%) | 405 (89.8%) |  |
| Abnormal | 98 (26.3%) | 34 (7.5%) |  |
| Not performed | 31 (8.3%) | 12 (2.7%) |  |
| **Race** |  |  | <0.001 |
| White | 202 (54.2%) | 395 (87.6%) |  |
| Black | 122 (32.7%) | 51 (11.3%) |  |
| Other | 49 (13.1%) | 5 (1.1%) |  |
| **Ethnicity** |  |  | <0.001 |
| Non-Hispanic | 279 (74.8%) | 310 (68.7%) |  |
| Hispanic | 84 (22.5%) | 141 (31.3%) |  |
| Other | 10 (2.7%) | 0 (0%) |  |
| **Family history of prostate cancer** |  |  | <0.001 |
| No | 309 (82.8%) | 381 (84.5%) |  |
| Yes | 12 (3.2%) | 69 (15.3%) |  |
| Not Reported | 52 (13.9%) | 1 (0.2%) |  |
| **Prostate Specific Antigen** | 6.79 [0.140–295] | 1.40 [0.100–54.9] | <0.001 |
| **Cancer** |  |  | <0.001 |
| No | 143 (38.3%) | 450 (99.8%) |  |
| Yes | 230 (61.7%) | 1 (0.2%) |  |
| **Grade Group** |  |  | <0.001 |
| GG1 (Gleason 3+3) | 105 (28.2%) | 1 (0.2%) |  |
| GG2 (Gleason 3+4) | 59 (15.8%) | 0 (0%) |  |
| GG3 (Gleason 4+3) | 26 (7.0%) | 0 (0%) |  |
| GG4 (Gleason 4+4) | 24 (6.4%) | 0 (0%) |  |
| GG5 (Gleason 4+5, 5+4, or 5+5) | 16 (4.3%) | 0 (0%) |  |
| Negative | 143 (38.3%) | 450 (99.8%) |  |
| **Observed microbial species** | 63.0 [29.0–94.0] | 63.0 [38.0–158] | 0.0115 |
| **Shannon diversity** | 3.57 [0.952–4.43] | 3.54 [1.90–4.59] | 0.625 |
| **Microbiome Score** | 0.370 [0.050–0.702] | 0.216 [0.0240–0.696] | <0.001 |

**Supplementary Table 2**. Multivariable logistic regression analysis of patients who underwent prostate biopsy

|  | **Clinically Significant (GG2+) Prostate Cancer vs. No Cancer** | | | **Clinically Significant (GG2+) Prostate Cancer** **vs. No Cancer/Grade 1 Cancer** | | | **Any Grade Prostate Cancer vs. No Cancer** | | |
| --- | --- | --- | --- | --- | --- | --- | --- | --- | --- |
|  | **(n=125 vs. 593)** | | | **(n=125 vs. 699)** | | | **(n=231 vs. 593)** | | |
| **Characteristic** | **OR***^1^* | **95% CI***^1^* | **p-value** | **OR***^1^* | **95% CI***^1^* | **p-value** | **OR***^1^* | **95% CI***^1^* | **p-value** |
| Age | 0.98 | 0.95–1.01 | 0.2 | 0.99 | 0.96–1.02 | 0.5 | 0.98 | 0.96–1.00 | 0.1 |
| Body mass index | 1.05 | 1.00–1.10 | 0.043 | 1.04 | 1.00–1.09 | 0.044 | 1.04 | 1.00–1.07 | 0.051 |
| Digital rectal examination |  |  |  |  |  |  |  |  |  |
| Normal | — | — |  | — | — |  | — | — |  |
| Abnormal | 5.7 | 3.07–10.7 | <0.001 | 4.14 | 2.40–7.13 | <0.001 | 3.58 | 2.16–5.96 | <0.001 |
| Not Performed | 0.3 | 0.06–1.03 | 0.086 | 0.41 | 0.09–1.26 | 0.2 | 0.31 | 0.12–0.77 | 0.016 |
| Race |  |  |  |  |  |  |  |  |  |
| White | — | — |  | — | — |  | — | — |  |
| Black | 1.84 | 0.95–3.57 | 0.069 | 1.52 | 0.84–2.75 | 0.2 | 1.81 | 1.11–2.95 | 0.018 |
| Other | 2.18 | 0.85–5.41 | 0.1 | 1.38 | 0.57–3.13 | 0.5 | 2.78 | 1.37–5.68 | 0.005 |
| Ethnicity |  |  |  |  |  |  |  |  |  |
| Non-Hispanic | — | — |  | — | — |  | — | — |  |
| Hispanic | 1.4 | 0.74–2.61 | 0.3 | 1.39 | 0.77–2.48 | 0.3 | 1.09 | 0.67–1.76 | 0.7 |
| Other | 1.5 | 0.15–10.2 | 0.7 | 1.16 | 0.14–6.31 | 0.9 | 1.1 | 0.23–5.07 | 0.9 |
| Family History Prostate cancer |  |  |  |  |  |  |  |  |  |
| N | — | — |  | — | — |  | — | — |  |
| Y | 0.38 | 0.06–1.37 | 0.2 | 0.34 | 0.05–1.21 | 0.2 | 0.53 | 0.20–1.20 | 0.2 |
| Not Reported | 3.08 | 1.33–7.08 | 0.008 | 2.16 | 1.03–4.41 | 0.037 | 3.91 | 2.01–7.80 | <0.001 |
| Prostate specific antigen | 1.17 | 1.11–1.23 | <0.001 | 1.14 | 1.10–1.19 | <0.001 | 1.18 | 1.12–1.23 | <0.001 |
| Microbiome Score^2^ | 3.5 | 1.87–6.90 | <0.001 | 2.87 | 1.58–5.51 | <0.001 | 2.79 | 1.80–4.41 | <0.001 |
| *^1^ OR, odds ratio; CI, confidence interval; ^2^ based on 0.25 threshold* | | | | | | | | | |

| **Characteristics** | **Any prostate cancer vs. no cancer** | **No prostate cancer and low risk prostate cancer vs. GG2+** |
| --- | --- | --- |
| Area under the curve (AUC) (Train/Validate) | 0.687 (95% CI 0.594–0.762, p<0.001) | 0.655 (95% CI 0.505–0.773, p<0.001) |
| AUC (10-fold cross validation) | 0.710 (95% CI 0.668–0.760, p<0.001) | 0.712 (95% CI 0.643–0.769). |
| Sensitivity | 82.70% | 84.80% |
| Specificity | 52.10% | 46.90% |
| Positive predictive value | 40.20% | 22.20% |
| Negative predictive value | 88.50% | 94.50% |

**Supplementary Table 3**. Operational characteristics for any prostate cancer grade and a comparison if low risk and GG1 prostate cancer were compared to GG2+ prostate cancer. The performance measures the PRIMUS scores’ ability for cancer detection.

**4.	Supplementary Figures**

**Supplementary Figure 1**. Microbiome diversity profiles. **(A)** We demonstrate a relative abundance plot where each line represents an individual group categorized by cancer status. The low-risk men were part of the SABOR screening cohort and did not have a prostate biopsy, whereas those in the no cancer, GG1, and GG2+ groups did undergo a prostate biopsy. Each color represents a different bacterial group noted in the figure key. **(B)** Shannon diversity scores display box and whisker plots to demonstrate the alpha diversity between groups. **(C)** A principal coordinates analysis illustrates the differences in beta diversity between groups, which are largely overlapping.

GG1, grade group 1; GG2+, greater than grade group 2 prostate cancer; SABOR, San Antonio Biomarkers of Risk

**Supplementary Figure 2**. Race, ethnicity, and microbiome diversity. **(A)** Shannon diversity scores display box and whisker plots to demonstrate the alpha diversity between groups. **(B)** A principal coordinates analysis illustrates the differences in beta diversity between groups, which are largely overlapping.

**Supplementary Figure 3**. Pipeline illustrating the development and analysis of the PRIMUS Microbiome Score for Prostate Cancer (MiSCaP). **(A)** Shows the steps from fastq files to the creation of the PRIMUS score. **(B)** Presents the relative risk profiles using linear discriminant analysis and the fold change on a log score, comparing cancer (red) and non-cancer (green). **(C)** Displays these relationships in a cladogram where microbes associated with cancer are grouped at the bottom of the graph in red, and microbes associated with non-cancer are grouped at the top.

PRIMUS, prostate cancer risk insight through microbiome understanding

**Supplementary Figure 4**. Youden’s Index to identify the ideal PRIMUS cut point. The current cut-off point on which this analysis is based is 0.25, with a second cut-off point for exploration at 0.3

PRIMUS, prostate cancer risk insight through microbiome understanding

**Supplementary Figure 5**. Operational Characteristics. **(a)** The ROC-AUC curve displays the training set (2/3, n=551) and validation set (1/3, n=273) model development, with associated histograms for cancer and no cancer. **(b)** Ten-fold cross-validation (n=824) during microbiome score development is compared with hold-out validation. **(c)** Calibration curves compare PRIMUS and Prostate Cancer Prevention Trial 2.0 risk for any prostate cancer. A line graph with 95% confidence intervals shows the predicted versus observed risk of prostate cancer diagnosis between PRIMUS and the PCPT 2.0 risk for overall prostate cancer detection. The top of the figure features a histogram displaying the distribution of PRIMUS scores and PCPT risk, indicating whether cancer was observed (top) or not (bottom). **(d and e)** The figures show histograms illustrating the distribution of PRIMUS scores across both the test-train and 10-fold validation cohorts. **(f)** The scatter plot depicts the correlation between PRIMUS model features obtained through cross-validation and the train-validate (hold-out) method. **(g)** The line graph displays the PRIMUS score by 10 groups (deciles) and prostate cancer risk, separated by any grade prostate cancer and GG2+ prostate cancer.

ROC, receiver operating characteristic; AUC, area under the curve; PRIMUS, prostate cancer risk insight through microbiome understanding; GG2+, greater than grade group 2 prostate cancer; PCPT,Prostate Cancer Prevention Trial risk calculator

**Supplementary Figure 6**. Microbiome scores correlate with prostate specific antigen (PSA) levels and provide benefit in prostate biopsy decision analysis (DCA). **(A)** Scatter plot comparing the log10-transformed PSA for a continuous variable with the PRIMUS Score (not transformed) and comparing cancer cases (red) to non-cancer cases. **(B)** We then calculate a decision analysis curve comparing PRIMUS and PSA for prostate biopsy decision-making. When the outcome is any grade prostate cancer, the DCA curves overlap, indicating minimal differences. **(C)** We calculated DCA curves with the outcome of clinically significant prostate cancer (GG2+), and PSA outperformed the PRIMUS score, especially at higher risk probabilities.

PRIMUS, prostate cancer risk insight through microbiome understanding; GG2+, greater than grade group 2 prostate cancer

**Supplementary Figure 7**. Incidence of any grade prostate cancer diagnosis to include grade group 1 stratified by PRIMUS score high (>0.25, red) compared to PRIMUS low (<0.25, blue). Time to any prostate cancer diagnosis. A Kaplan‒Meier plot showed decreased prostate cancer-free survival in patients with a baseline PRIMUS score of 0.25 or greater (hazard ratio [HR] 3.97, 95% CI 2.14–7.36, *P*<0.001).

PRIMUS, prostate cancer risk insight through microbiome understanding

**Supplementary Figure 8**. Polyketide synthase (PKS) as an added variable to the PRIMUS score. We demonstrate an incidence plot showing the new diagnosis of cancer groups by high or low PRIMUS score stratified by PKS status (colibactin gene positive by PCR testing).

PRIMUS, prostate cancer risk insight through microbiome understanding; PKS, polyketide synthase; PCR, polymerase chain reaction

**Supplementary Figure 9**. Microbiome scores generated from different bacterial variable regions are significantly correlated. The scatter plot displays a single patient sample sequenced using variable regions V1-V2 (x-axis) and V3-V4 (y-axis). The scores are significantly correlated (Pearson correlation = 0.77, p<0.001).

**Supplementary Figure 10**. Alterations in the microbiome score with previous antibiotic use. The box and whisker plots show that the “No cancer” groups are similar and the “cancer” groups have similar PRIMUS scores despite the use of previous antibiotics.

PRIMUS, prostate cancer risk insight through microbiome understanding

**Supplementary Figure 11**. Predicted metabolic functional analysis between high and low PRIMUS scores. **(a)** The top associated results using the relative fold change and the PICRUSt similarity pathways are Lung Cancer and Colon Cancer. (B) Lung cancer. Using an external cohort [27], we used the PRIMUS score to determine if the score is associated with lung cancer, separated by sex, and displayed as box plots comparing those with and without lung cancer. (C) Colorectal cancer. We compared another external cohort [28] stratified by sex, and using box plots of the PRIMUS score, we compared those with and without colorectal cancer

PRIMUS, prostate cancer risk insight through microbiome understanding

**Supplementary Figure 12**. Investigation of previous studies on Mendelian randomization. **(A)** A chart showing the bacteria identified in previous studies [24, 25] as potentially causally linked to prostate cancer, indicating whether they are associated with increased or decreased risk, and which study identified them. **(B)** Shows bacteria clustered by cohort and grade, and **(C)** shows unsupervised clustering, both without distinct patterns. **(D)** is a t-distributed stochastic neighbor embedding (t-SNE) plot and **(E)** is a Uniform Manifold Approximation and Projection (UMAP), both do not show clustering with cohort or grade of cancer.

**Supplementary Figure 13.** External validation for calculating the PRIMUS score from patients with inflammatory bowel disease noted modifiability in a high-risk cohort. (**A**) PRIMUS Score in Inflammatory Bowel disease. We obtained 16S rRNA gene DNA sequences from external studies of the Human Microbiome Project 2 (HMP2) and calculated the PRIMUS score. The box and whisker plots show no difference in PRIMUS scores in females with IBD (top left, Student’s t-test, *P*=0.98) and a significantly higher PRIMUS score in men with IBD (top right, *P*=0.0024). (**B**) PRIMUS score in patients with IBD before and after a randomized clinical trial using Fecal Microbiota Transplant (FMT). The PRIMUS score was calculated using the study data and showed lower microbiome scores in donor stool samples (*P*<0.001). A comparison of the microbiome scores 25–35 days post-FMT revealed that the intervention group had PRIMUS scores that resembled those of the donors (*P*=0.12). In contrast, the PRIMUS score of the placebo group was significantly higher than that of the intervention group (*P*=0.002). (**C**) The PRIMUS score was plotted continuously with a smooth line, showing separation 20 days post-FMT with elevation starting after the end of transplantation treatment on day 35.

PRIMUS, prostate cancer risk insight through microbiome understanding

**5. Supplementary References**

[1] Liss MA, Al-Bayati O, Gelfond J, Goros M, Ullevig S, DiGiovanni J, et al. Higher baseline dietary fat and fatty acid intake is associated with increased risk of incident prostate cancer in the SABOR study. Prostate Cancer Prostatic Dis. 2019;22:244–51. <https://doi.org/10.1038/s41391-018-0105-2>.

[2] Siegel RL, Miller KD, Wagle NS, Jemal A. Cancer statistics, 2023. CA Cancer J Clin. 2023;73:17–48. <https://doi.org/10.3322/caac.21763>.

[3] US Preventive Services Task Force, Grossman DC, Curry SJ, Owens DK, Bibbins-Domingo K, Caughey AB, et al. Screening for prostate cancer: US Preventive Services Task Force recommendation statement. JAMA. 2018;319:1901–13. <https://doi.org/10.1001/jama.2018.3710>.

[4] Miller KD, Nogueira L, Devasia T, Mariotto AB, Yabroff KR, Jemal A, et al. Cancer treatment and survivorship statistics, 2022. CA Cancer J Clin. 2022;72:409–36. <https://doi.org/10.3322/caac.21731>.

[5] Carlsson SV, Kattan MW. Prostate cancer: personalized risk—stratified screening or abandoning it altogether? Nat Rev Clin Oncol. 2016;13:140–2. <https://doi.org/10.1038/nrclinonc.2016.11>.

[6] McBride CM, Puleo E, Pollak KI, Clipp EC, Woolford S, Emmons KM. Understanding the role of cancer worry in creating a “teachable moment” for multiple risk factor reduction. Soc Sci Med. 2008;66:790–800. <https://doi.org/10.1016/j.socscimed.2007.10.014>.

[7] Qin J, Li R, Raes J, Arumugam M, Burgdorf KS, Manichanh C, et al. A human gut microbial gene catalogue established by metagenomic sequencing. Nature. 2010;464:59–65. <https://doi.org/10.1038/nature08821>.

[8] Turnbaugh PJ, Ley RE, Hamady M, Fraser-Liggett CM, Knight R, Gordon JI. The human microbiome project. Nature. 2007;449:804–10. <https://doi.org/10.1038/nature06244>.

[9] Bolger AM, Lohse M, Usadel B. Trimmomatic: a flexible trimmer for Illumina sequence data. Bioinformatics. 2014;30:2114–20. <https://doi.org/10.1093/bioinformatics/btu170>.

[10] Caporaso JG, Kuczynski J, Stombaugh J, Bittinger K, Bushman FD, Costello EK, et al. QIIME allows analysis of high-throughput community sequencing data. Nat Methods. 2010;7:335–6. <https://doi.org/10.1038/nmeth.f.303>.

[11] Magoč T, Salzberg SL. FLASH: fast length adjustment of short reads to improve genome assemblies. Bioinformatics. 2011;27:2957–63. <https://doi.org/10.1093/bioinformatics/btr507>.

[12] Kuczynski J, Stombaugh J, Walters WA, González A, Caporaso JG, Knight R. Using QIIME to analyze 16S rRNA gene sequences from microbial communities. Curr Protoc Bioinformatics. 2011; Chapter:10.7.1–10.7.20;Chapter:10.7.1–10.7.20. <https://doi.org/10.1002/0471250953.bi1007s36>.

[13] Langmead B, Salzberg SL. Fast gapped-read alignment with Bowtie 2. Nat Methods. 2012;9:357–9. <https://doi.org/10.1038/nmeth.1923>.

[14] R-Forge. Vegan: Community Ecology Package; 2010, http://vegan.r-forge.r-project.org/.

[15] Langille MGI, Zaneveld J, Caporaso JG, McDonald D, Knights D, Reyes JA, et al. Predictive functional profiling of microbial communities using 16S rRNA marker gene sequences. Nat Biotechnol. 2013;31:814–21. <https://doi.org/10.1038/nbt.2676>.

[16] Benjamini Y, Drai D, Elmer G, Kafkafi N, Golani I. Controlling the false discovery rate in behavior genetics research. Behav Brain Res. 2001;125:279–84. <https://doi.org/10.1016/s0166-4328(01)00297-2>.

[17] Jeng LL, Moore RM, Jr., Kaczmarek RG, Placek PJ, Bright RA. How frequently are home pregnancy tests used? Results from the 1988 national maternal and infant health survey. Birth. 1991;18:11–3. <https://doi.org/10.1111/j.1523-536x.1991.tb00046.x>.

[18] Segata N, Izard J, Waldron L, Gevers D, Miropolsky L, Garrett WS, et al. Metagenomic biomarker discovery and explanation. Genome Biol. 2011;12:R60. <https://doi.org/10.1186/gb-2011-12-6-r60>.

[19] Kairalla JA, Coffey CS, Muller KE. GLUMIP 2.0: SAS/IML software for planning internal pilots. J Stat Softw. 2008;28:1. <https://doi.org/10.18637/jss.v028.i07>

[20] Ruopp MD, Perkins NJ, Whitcomb BW, Schisterman EF. Youden Index and optimal cut-point estimated from observations affected by a lower limit of detection. Biom J. 2008;50:419–30. <https://doi.org/10.1002/bimj.200710415>.

[21] Vickers AJ, Van Calster B, Steyerberg EW. Net benefit approaches to the evaluation of prediction models, molecular markers, and diagnostic tests. BMJ. 2016;352:i6. <https://doi.org/10.1136/bmj.i6>.

[22] Gelfond JA, Hernandez B, Goros M, Ibrahim JG, Chen MH, Sun W, et al. Prediction of future risk of any and higher-grade prostate cancer based on the PLCO and SELECT trials. BMC Urol. 2022;22:45. <https://doi.org/10.1186/s12894-022-00986-w>.

[23] Integrative HMP (iHMP) Research Network Consortium. The Integrative Human Microbiome Project: dynamic analysis of microbiome-host omics profiles during periods of human health and disease. Cell Host Microbe. 2014;16:276–89. <https://doi.org/10.1016/j.chom.2014.08.014>.

[24] Long Y, Tang L, Zhou Y, Zhao S, Zhu H. Causal relationship between gut microbiota and cancers: a two-sample Mendelian randomisation study. BMC Med. 2023;21:66. <https://doi.org/10.1186/s12916-023-02761-6>.

[25] Yin Z, Liu B, Feng S, He Y, Tang C, Chen P, et al. A large genetic causal analysis of the gut microbiota and urological cancers: A bidirectional Mendelian randomization study. Nutrients. 2023;15:4086. <https://doi.org/10.3390/nu15184086>.

[26] Crothers JW, Chu ND, Nguyen LTT, Phillips M, Collins C, Fortner K, et al. Daily, oral FMT for long-term maintenance therapy in ulcerative colitis: results of a single-center, prospective, randomized pilot study. BMC Gastroenterol. 2021;21:281. <https://doi.org/10.1186/s12876-021-01856-9>.

[27] Zeller G, Tap J, Voigt AY, Sunagawa S, Kultima JR, Costea PI, et al. Potential of fecal microbiota for early-stage detection of colorectal cancer. Mol Syst Biol. 2014;10:766. <https://doi.org/10.15252/msb.20145645>.

[28] Haberman Y, Kamer I, Amir A, Goldenberg S, Efroni G, Daniel-Meshulam I, et al. Gut microbial signature in lung cancer patients highlights specific taxa as predictors for durable clinical benefit. Sci Rep. 2023;13:2007. <https://doi.org/10.1038/s41598-023-29136-4>.
